# Why do people become health workers? Analysis from life histories in 4 post‐conflict and post‐crisis countries

**DOI:** 10.1002/hpm.2485

**Published:** 2018-01-12

**Authors:** Sophie Witter, Haja Wurie, Justine Namakula, Wilson Mashange, Yotamu Chirwa, Alvaro Alonso‐Garbayo

**Affiliations:** ^1^ ReBUILD Consortium Queen Margaret University Musselburgh, Edinburgh UK; ^2^ ReBUILD Consortium and College of Medicine and Allied Health Sciences University of Sierra Leone Freetown Sierra Leone; ^3^ ReBUILD and Department of Health Policy, Planning and Management, Makerere School of Public Health New Mulago Hospital Complex Kampala Uganda; ^4^ ReBUILD and Centre for International Health Policy and Biomedical Research and Training Institute, Health Systems unit Harare Zimbabwe; ^5^ ReBUILD and Department of International Public Health and Liverpool School of Tropical Medicine, Pembroke Place Liverpool UK

**Keywords:** Health workers, motivation, post‐conflict, post‐crisis

## Abstract

While there is a growing body of literature on how to attract and retain health workers once they are trained, there is much less published on what motivates people to train as health professions in the first place in low‐ and middle‐income countries and what difference this makes to later retention. In this article, we examine patterns in expressed motivation to join the profession across different cadres, based on 103 life history interviews conducted in northern Uganda, Sierra Leone, Cambodia, and Zimbabwe. A rich mix of reported motivations for joining the profession was revealed, including strong influence of “personal calling,” exhortations of family and friends, early experiences, and chance factors. Desire for social status and high respect for health professionals were also significant. Economic factors are also important—not just perceptions of future salaries and job security but also more immediate ones, such as low cost or free training. These allowed low‐income participants to access the health professions, to which they had shown considerably loyalty. The lessons learned from these cohorts, which had remained in service through periods of conflict and crisis, can influence recruitment and training policies in similar contexts to ensure a resilient health workforce.

## 1 INTRODUCTION

The makeup of the health workforce is a key factor driving health sector performance, but we continue to face major challenges in recruiting and retaining health workers of the right kind and in the right places to drive progress towards Universal Health Coverage and to meet Sustainable Development Goal 3. The recent global strategy on human resources for health^1^ highlighted that at least 18 million additional health workers in low‐ and middle‐income countries (LMICs) are needed to provide high and effective coverage of the broad range of health services necessary to ensure healthy lives for all. Investing in the health workforce also bears potential for important economic returns.^2^

While there is a growing body of literature on how to attract and retain health workers once they are trained,^3^, ^4^, ^5^, ^6^ there is much less written on how to attract people to join the health professions in the first place in LMICs, what motivates them to join the health professions and what difference their motivations to join might make to health workers' retention. Various health labour market frameworks have been developed to analyse supply and demand for health workers (for example, Vujicic and Zurn^7^ and Witter et al^7^, ^8^). These typically view the demand of health workers for training as a function of the number of applicants, tuition costs, and expected wages. Different psychological profiles and the more varied factors which motivate people to apply to join the profession are somewhat lost in these frameworks. However, the motivations to join may be of significance not only to entry but also for subsequent careers.

This article stems from a research programme looking at lessons on reconstructing health systems post‐conflict. Four countries were selected to represent different contexts and distance in time from conflict, so that the post‐conflict trajectories could be studied: Zimbabwe, which experienced economic and political crises, peaking in 2008; northern Uganda, which emerged from decades of Lord's Resistance Army–sponsored war in 2006; Sierra Leone, whose civil war ended in 2002 and was recently affected by an Ebola outbreak in 2014 to 2015; and Cambodia, where a comprehensive peace post‐Khmer Rouge began in 1999. Amongst other projects, we examined changing incentive environments for health workers.^9^ Many fragile and conflict‐affected states struggle to maintain an adequate and balanced health workforce.^10^, ^11^ Within this project, we used life histories to examine the experiences of health workers during different phases of their career and their country's history. Different themes emerged, including motivation to join the health profession.

In this article, we examine patterns in expressed motivation to join the profession across different settings and cadres, bearing in mind that these participants were selected for having stayed in service during and after conflict periods. An underlying question is whether their motivational profile helps in part to explain their retention and what that implies for human resources for health policies in the health systems studied. All 4 countries have faced chronic challenges in retaining staff, especially in rural areas, and have had to build their health workforce back up after the shocks of conflict, epidemics, and economic collapse.

## 2 METHODS

A mixed methods study on health worker incentives was designed, using both retrospective and cross‐sectional tools, one of which was life histories with health workers in 4 countries. The objective of the overall research was to understand changing health worker incentives and their policy implications in the post‐conflict and post‐crisis period.^9^

Life histories were deployed to explore health workers' perceptions and experiences of their working environment, how it has evolved and factors which would encourage or discourage them from staying in post in remote areas and being productive. They were encouraged to produce visual aids, such as timelines. Through their lives and experiences, we sought to obtain understanding of the evolution of the health system and the different processes related to the work environment. Their lived experiences provided us with a personal perspective on the effectiveness and intended as well as unintended consequences of human resource policies and their evolution.^12^

These life histories were conducted between October 2012 and December 2013 in the 4 settings with health workers meeting specific criteria (including length of service in the area, to capture experiences of conflict and post‐conflict periods) in selected health care facilities in the study areas using an open‐ended topic guide. The topic guide covered the following areas:

- How and why they became health workers;
- Their career path since, and what influenced it, including the role of gender;
- What motivates/discourages them to work in rural areas and across different sectors;
- Challenges they face in their job and how they cope with them;
- Conflict related challenges and how they coped;
- Their career aspirations;
- Their knowledge and perceptions of recent and current incentives.

The profile of the participants is shown in Table 1. They represent the mix of staff actually found on the front line in health centres, which tends to be dominated by mid‐level cadres, who are largely female.^13^

**Table 1:** Summary of life histories

Topic	Cambodia	Sierra Leone	Uganda	Zimbabwe
Site selection	Six provinces (covering all 4 ecological regions)—one district from each, including urban, rural, and those with more or less external support	Four districts (covering all main regions, including urban and rural/hard to reach and areas of varied socio‐economic status)	Three districts in Acholi subregion—most conflict‐affected area	Two provinces—one well served and one under‐served; three districts including urban, mixed and rural
Sectors included	Public sector only	Public sector only	65% public; 35% PNFP (private not‐for‐profit—largely mission sector)	9 from the government sector; 14 from the municipality; 2 from the rural district councils; 6 from the mission sector; and 4 from the private sector (but these were public staff working part‐time for private facilities)
Health workers interviewed	Total: 19	Total: 23	Total: 26	Total: 35
	By cadre: 4 doctors; 1 medical assistant; 8 midwives; 6 nurses	By cadres: State registered nurse 1, staff midwife 4, public health sister/district health sister 2, M&E officer 1, CHO 2, senior CHO5, matron 5, medical superintendent 2 and senior specialist 1	By cadres: 2 clinical officers; 15 nurses; 2 nursing assistants 3 midwives; 2 others	By cadres: 2 doctors; 21 midwives; 9 nurses; 3 environmental health practitioners
	By gender: 14 f; 5 m	By gender: 12 f/11 m	By gender: 19f; 7 m	By gender: 32 f; 3 m
	Age range: 24‐53	Age range: 36‐65	Age range: 30‐60	Age range: 31‐65

Thematic analysis using manual coding, NVIVO (and ATLAS Ti in Uganda) was conducted on transcribed (and sometimes translated) texts. The analysis started from the main themes of the life history topic guide, but codes were then developed inductively from the responses of the health workers in each context. Here, we have compared across the 4 settings to identify the cross‐cutting major elements which motivated respondents to join their professions. Other aspects of the interviews have been analysed elsewhere by country and across countries^14^

Ethical approval was gained from each country ethics board and the relevant UK university. The interviews were tape‐recorded and noted after gaining permission from the participants. The interviews took place in a private place acceptable to the interviewee, such as their office. The identity of the participants has been protected during analysis and reporting.

### 2.1 Limitations

The health workers interviewed represent “positive deviants”—those who stayed in service during difficult times for the country (or subregion, in the case of Uganda)—and cannot therefore be taken to represent the wider health workforce. However, the autoethnographic method^15^ can provide rich insights into experiences which can productively inform health workforce planning. They can indicate some common motivational factors; although with these qualitative methods and given the range of contexts and health worker types, we cannot provide magnitudes of salience for different factors.

## 3 FINDINGS

For the staff we interviewed, there was a remarkable commonality of reasons given for joining the profession. Across all 4 settings, personal calling was the mostly commonly cited, closely followed by personal connections—the influence of family and significant others, which was exercised in a variety of ways. The status of health professionals was also a shared attractive feature, as perceived by participants. In addition, some more pragmatic reasons for joining were expressed, including living near to health facilities where training could be undertaken, economic factors relating to training costs and posts, crises which impelled people to seek early earning opportunities, educational factors which pushed or pulled candidates into training, and practical issues relating to security. There were often connections between the different motivating factors.

### 3.1 Personal calling

Many of the participants reported that they had desired to be a health professional since their childhood. A common theme behind this was the desire to serve their community, linked to a caring personality.

“Reason for choosing this career because I love it and can help people in my community” (Cambodia)

“During my primary school days, there was a hospital managed by the Chinese by then...During that era I came to realise that serving people through that way appealed to me” (Sierra Leone)

“It was a calling and feeling of wanting to serve people, so I thought that if I am trained I can also come and save the life of my people” (Uganda)

“I came to work as a nurse because I volunteered to work in the health field during the war; I had a passion for the job” (Zimbabwe)

This caring ethic often has religious roots but can also be triggered by personal backgrounds, which give people empathy into poor health and access issues. Many of the cadres, such as nurses, are highly feminised and the caring role is one with strong gender connotations in many of these contexts.

“I came from school directly to nursing because nursing was my career….I had passion for people so I decided to be a nurse because I wanted to save humanity and since I am a Christian I thought this way. Because the healing ministry was one of the focus when our Lord and Jesus Christ was on earth so ….that was my focus to save humanity and to help people” (Zimbabwe)

“I chose nursing as a career, so that I can help especially my family. Looking at the area I came from, medical facilities in those areas is a bit poor, for instance from my village to where we have the health centre is over ten miles” (Sierra Leone)

### 3.2 Family connections and role models

Family members are very influential in career decisions, both directly through instruction and advice and indirectly through family illnesses and role modelling. Family influences seemed to play a stronger role for women than men.

“My Mother too wanted me to do nursing…She made all the arrangements that I should do nursing because she had wanted to do it but she did not do it.…that's what made me to do it….and then with the great help of my uncle he decided that I should be in the nursing profession” (Sierra Leone)

“Before my father died in 1972, he used to tell me, ‘you are so calm, you are fit to be a nurse and he could tell people that this one can be a very good nurse’” (Uganda)

“I just wanted to be a nurse from way back; my sister was also a nurse so I admired her so much” (Zimbabwe)

Decisions are often influenced by family members, sometimes even against the preferences of the young person.

“My relatives love this subject and they pushed me to study this subject” (Cambodia)

Having close relatives with health problems was reported to have motivated the decisions to apply for health studies, in some cases, to provide care for them directly or to avoid having to pay for it.

“Actually I am interested in this field because when I was a student, my brother was often sick. We spent a lot of money to take care of him. My relatives mostly are teacher and soldier. Thus, I thought that no one could help us besides ourselves” (Cambodia)

In many circumstances, role models knowingly or unknowingly mentored the participants to become health workers. For example, positive attitudes of health workers as they cared for the participants' relatives had a lasting impression on some participants during their childhood and later translated into a need to emulate this good work “pay back to society” and provide a high standard of care by joining the health workforce. On the contrary, those who had experienced poor attitudes from health workers also felt compelled in some cases to join the health sector to change this poor quality of care.

“What drove me to become a health worker is the personality of elders who were working in our hospital of Kitgum … I got to like the way they used to handle the patients and it motivated me to become like them in future” (Uganda)

### 3.3 Status of professionals

Social status and esteem accorded to the health professions was another important cross‐cutting theme, which comingled with pay and the desire to serve:

“I learnt that doctor had good income and respect from people in the community. I can also help people” (Cambodia)

“Those gentlemen [health workers] from my home area were highly respected ... that was the reason why I went for health training” (Uganda)

One facet of social esteem was the dress code of the nurses and the way in which they presented themselves. Respect for the medical profession in society and admiration of health professional mannerisms and the uniform (particularly for nurses) was very appealing to some (only in Cambodia was the allure of the uniform not mentioned). The work ethic of health professionals also fuelled the aspiration of some to join the medical field.

“I loved it. When I was younger I used to admire the young nurses in our chiefdom headquarter town, the way they dress, the way they walk, the way they talk, so I said if I could be a nurse it will be better for me” (Sierra Leone)

“Those gentlemen could put on smartly... and that was the reason I went for health training” (Uganda)

“I used to adore nurses in their white uniforms since I was young so I came to train as a nurse in 1995 up to May 1999” (Zimbabwe)

### 3.4 Economic factors

Decisions to join the health profession were also based on economic perspectives. In some cases, families advised their children to choose a health profession as these were perceived to be better paid than others:

“They suggested to me for applying an exam in order to become a teacher and a midwife. I passed both exams. However, we thought that the salary for teachers is too little. On the other hand, midwife can be better. So, I decided to become a midwife” (Cambodia)

In Uganda, one respondent reported that they were motivated by the incentives provided to the health workers at the time. These included accommodation and transport.

“The health workers were accommodated well, they had means of transport, I thought it was good work and that was the reason why I went for health training” (Uganda)

Some participants, particularly these with more economic problems and hence in urgent need to earn a salary, reported having selected their health profession according to the time required to complete the studies, as this secondary midwife reported:

“Actually, I wished to become a Medical Assistant, but it took longer time for study; and my family was very poor (laugh)… I spent a shorter time to complete my study and then went back to work and took care of my family” (Cambodia)

The ability to earn quickly while also training was an attraction for those from poorer households.

“I liked nursing because if someone is trained he/she is given a place to work unlike whereby someone has to struggle looking for a job. We were given uniforms and accommodation for free and we also used to get our salaries the first month you are enrolled” (Zimbabwe)

Financial costs of training and constraints were also mentioned as factors in choice of course, for example, not being able to afford the tuition fees and therefore opting for the tuition‐free paramedical school in Sierra Leone.

“But ehmm financially that was not possible, the support I needed was not there and paramedical school was offering cost free education at that moment. So I had to try my luck and fortunately for me immediately I applied the very year I was admitted into the school” (Sierra Leone)

The nature of nurse training in Zimbabwe, particularly the lack of tuition fees, access to monetary allowances at enrolment and the fact that after training one was assured of a posting, were very strong motivations for individuals' decisions to become health workers, especially those from lower income households.

“We were paying fees and as a person who was coming from a poor family, I was not managing to pay the fees… dropped out of training after getting a place to train as a nurse” (Zimbabwe)

Some started their careers working for nongovernmental organisations, such as the Red Cross. Nongovernmental organisations offered good support to health workers with accommodation, financial support for utility bills, etc, which given the relatively precarious economic situation of young professionals, represented an important incentive:

“At the first time in 2004, I was a contracted employee. I was supported by Cambodia Red Cross at the beginning. They rented a house and other stuff for me. They helped me pay for electricity and water utility… [] That is the reason why I could work in the health centre. Otherwise, I could not continue working here” (Cambodia)

A crisis in the family and the loss of a family head was also a trigger in some cases.

“So when I reach form 5, I said ok if I sit my O Level, I will like to go to the university. But my father died and my mum didn't have much, so he [referring to uncle] was like advising: ‘Why can't you do nursing [which was free then] instead of going to the university because definitely you cannot afford it” (Sierra Leone)

“Before I completed Senior 6, the war became serious. My father told me to come home and wait until the situation was calm. However, the soldiers came and shot my father, he was shot when me and my mother were seeing, so that was the end of the story. So from there, my brother …he told me ‘now you cannot proceed, you join a nursing school”’ (Uganda)

Others decided based on the perceived labour market demand for specific disciplines and specialties:

“I studied general medical doctor [lung diseases] because [there are] only a few of doctors in this field” (Cambodia)

### 3.5 Educational background

Another factor that influenced the decision to become a health worker was the subjects studied in secondary school. In general, having done science subjects predisposed individuals to join the medical profession.

“I studied the sciences; I thought that was a very good opportunity for me to do so, that is how I came into health” (Sierra Leone)

The health profession was perceived to be more flexible in relation to education backgrounds. Hence, some of those who were unable to advance further in their formal education as a result of conflict saw health training as an opportunity to continue their education and get a job without having to present very high qualifications, such as a university degree.

“I completed senior 4 because of the war and during that time (1987) the war of Kony was very serious here that I could not continue with studies ... that was also another reason why I decided to join” (Uganda)

However, some choices were forced onto candidates by failing to quality for medicine, as in the case of this environmental health practitioner:

“I wanted to do medicine but my points were not sufficient so I enrolled to do Environmental Health Sciences” (Zimbabwe)

### 3.6 Proximity to facilities

Location also emerges as an important factor, especially for mid‐level cadres, whose access to training can be through local volunteer and on the job training, followed by more formal training. In Uganda, for example, the proximity of convents, health facilities, schools, and medical training institutions was reported to play a major role for many of the respondents in their decision to join the health workforce.

“When the hospital was established, it was announced for volunteer medical staff. Because I knew the village chief well, my father asked for my name as volunteer” (Cambodia)

Other participants stated that they joined the medical field because they grew up within an area with a mission hospital and a nurse training school. Proximity to the hospital increased interaction with nurses and the admiration for their profession. Several participants grew up, attended school, and trained at these mission training hospitals and on completion worked at these posts.

“I grew up in this area and I came to church here when I was very young so I was inspired by the way sisters and nurses attend to patients” (Zimbabwe)

Again, some gendered themes emerged. For example, in Cambodia, staying close to the family was considered important for many female participants, who sometimes chose their profession to ensure they were close to their relatives.

“According to my family as I am a woman, they didn't want me to go far away from home. So they suggested to me to apply for an exam in order to become a teacher and a midwife” (Cambodia)

In this specific conflict, this may have acquired even more importance as during the Khmer Rouge government people were often taken away from their home environments and sometimes families were separated as a strategy to disintegrate social networks. In reaction, being near their home place probably represented an even stronger consideration in the post‐conflict period.

### 3.7 Chance events and lack of (or worse) alternatives

Some participants joined the health profession due to lack of alternatives in the job market or being unable to enter their chosen career.

“I wanted to be a police woman but I was short changed by my height… I joined nursing as it had a name in the community” (Zimbabwe)

In Cambodia, one reason given for choosing the health field was to avoid being recruited by the army, which was particularly an issue before the end of the conflict.

“The reason why I chose the medical area because in the past, there was war, and we were better off to be doctor than to be soldiers going out to war” (Cambodia)

In a few cases, the entry was by chance—for example, by responding to an advert but without advance planning or knowledge of the profession.

“The political situation was not conducive to staying in the rural areas in 1983, we then came to town. That is when I came across a MoHCW advert and I applied and was shortlisted and attended an interview at PMD's offices. I was successful and was selected to go and train” (Zimbabwe)

## 4 DISCUSSION

Our findings suggest that for this cohort at least, motivation to join the health care profession was heavily influenced by personal factors—not just a sense of “calling” but also early exposure to health work through proximity and family factors, and also the strong influence of family members and other role models. These are less amenable to policy action in terms of attracting staff but are of interest when we consider that our cohort had remained in service through very difficult times in terms of conflicts and crises. In a related report on the effects of the Ebola crisis in Sierra Leone, patriotism also emerged as a motivating factor for staff.^15^ These findings reinforce the conclusion that recruitment strategies should focus on staff with strong intrinsic motivation, especially to improve retention in hard to serve areas and over periods requiring resilience in the health system. This is especially important in times of conflict and crisis, when pay is erratic, working conditions are difficult and formal structures of promotion and recognition do not function well.^17^ Volunteering, for example, was a common entry for lower‐level staff, and these staff can be provided with formal training that enables them to benefit from promotion higher in the ranks at a later stage. Their commitment to service in their local area is often higher. However, measures are needed to ensure that quality of care is maintained and that volunteering is not used as a way to keep staff unpaid for long periods (as has happened in the case of Sierra Leone^18^); this leads to unwelcome side effects in the form of informal charges.

The findings also highlight some important themes related to professional status, which should be considered in policy circles. For example, across all 3 African settings, the importance of uniform was highlighted—the uniforms of health workers symbolised the status of the professions for this cohort. It may be important to consider how that plays out now, in an era when respect for health workers is eroding in some contexts, with hostile media reports about absenteeism and pilfering (for example, in Uganda) and lack of trust between health workers and the communities highlighted in the face of an epidemiological crisis (eg, the Ebola outbreak in Sierra Leone).^16^ Taking action to maintaining “brand image” may be a low‐cost but high‐gain strategy for sectoral managers and leaders.

At the same time, some important features emerge in relation to the economic motivations. Many of our respondents came from poor households and chose the health professions because training was supported at that time and enabled early earning. They then remained in post over a long period, in some cases in their original training site. Training policies should focus on offering good access for these less advantaged local students if they are seeking strong retention and loyalty to the sector.

Gendered expectations also emerged as a theme—both in terms of encouraging entry to “caring” professions and also in relation to staying in service in local areas. The health workforce and its roles are highly gendered in most countries, including these^13^ and health workforces will continue to be feminised, especially at lower levels. Ensuring gender equity for staff and gender responsive services for users will involve fostering dialogue and action to support change within institutions, households, and at policy level.

Few differences were noted by cadre and sector; although in some contexts, such as Uganda, the PNFP institutions took on a large part of the training activities, particularly in the northern regions where our interviews took place.^19^ This was also true in Zimbabwe where faith‐based organisations, by running most of the health professional education institutions, were able to select and retain the best performers.^20^

These findings are likely to apply beyond the conflict‐affected contexts in which they were collected but do of course reflect their place and time. For example, in the post‐conflict period, the workforce is often swelled rapidly through training of low‐level cadres who take on more responsibilities until longer term training of high‐level cadres can be completed, leading to an unhappy transition when these later cohorts take over.^21^, ^22^ Later policies can be less supportive of entry for lower income candidates—for example, paramedical and nursing training is no longer universally free in many of these contexts, such as Sierra Leone and Uganda; although in Zimbabwe, it remains free, even for post‐basic training, and students are taken on payroll when they start their training. It is likely that factors influencing staff to join the professions now are different, although some underlying factors, such as psychological profiles and the search for professional esteem and a reliable income, are likely to remain constant. These influence the desire not only to join but also to stay.^17^, ^22^, ^23^, ^24^, ^25^

The literature exploring motivation to join the health professions in LMICs is limited, but the studies which focus on professional motivation more generally also find similar themes such as self‐motivation and desire to serve the community and obtain respect (for example, for outgoing nurse trainees in India).^26^ A recent labour market survey from another post‐conflict context—Timor Leste—also found that the vast majority of all cadres (98% of doctors and nurses and 97% of midwives) reported that they selected health care as a career to help people.^27^ In the context of a newly independent nation with a young cohort of health workers, a high degree of idealism is to be expected.

## 5 CONCLUSIONS

Our life histories with a range of health staff in 4 conflict‐ and crisis‐affected settings find a rich mix of reported motivations for joining the profession, including a strong influence of “personal calling,” the exhortations of family and friends (including positive role models but also the pressure of gendered expectations and the need to support family members), early experiences of the health care sector through family illness, and chance factors such as proximity to facilities. Desire for social status and high respect for health professionals is influenced by factors such as smart presentation and uniforms. Economic factors are also important—not just perceptions of future salaries and job security but also more immediate ones, such as low cost or free training, the ability to earn while learning, making savings on treatment of sick relatives, and the availability of supportive allowances. These allowed low income participants to access the health professions, to which they are then likely to show considerably loyalty. The lessons learned from these cohorts, which had remained in service through periods of conflict and crisis in Uganda, Zimbabwe, Sierra Leone, and Cambodia, can influence recruitment and training policies in similar contexts to ensure a resilient health workforce. They emphasise the importance of attracting candidates with prosocial motivations and local ties but then also reinforcing and maintaining them through development of the professional brand, for example.
